# Severe hepatic encephalopathy in a patient with liver cirrhosis after administration of angiotensin-converting enzyme inhibitor/angiotensin II receptor blocker combination therapy: a case report

**DOI:** 10.1186/1752-1947-4-141

**Published:** 2010-05-19

**Authors:** Sabine Oertelt-Prigione, Andrea Crosignani, Maurizio Gallieni, Emanuela Vassallo, Mauro Podda, Massimo Zuin

**Affiliations:** 1 Institute of Gender in Medicine, Charité - Universitätsmedizin, Luisenstrasse 65, 10115 Berlin, Germany; 2 Division of Internal Medicine and Liver Unit, Department of Medicine, Surgery and Dentistry, San Paolo Hospital School of Medicine, University of Milan, via di Rudiní 8, 20142, Milan, Italy; 3 Nephrology and Dialysis Unit, San Paolo Hospital, University of Milan, via di Rudiní 8, 20142, Milan, Italy; 4 Department of Internal Medicine, IRCCS Istituto Clinico Humanitas, via A Manzoni 113, 20089 Rozzano, Italy

## Abstract

**Introduction:**

A combination therapy of angiotensin-converting enzyme inhibitors and angiotensin II receptor blockers has been used to control proteinuria, following initial demonstration of its efficacy. However, recently concerns about the safety of this therapy have emerged, prompting several authors to urge for caution in its use. In the following case report, we describe the occurrence of a serious and unexpected adverse drug reaction after administration of a combination of angiotensin-converting enzyme inhibitors and angiotensin II receptor blockers to a patient with nephrotic syndrome and liver cirrhosis with severe portal hypertension.

**Case presentation:**

We administered this combination therapy to a 40-year-old Caucasian man with liver cirrhosis in our Hepatology Clinic, given the concomitant presence of glomerulopathy associated with severe proteinuria. While the administration of one single drug appeared to be well-tolerated, our patient developed severe acute encephalopathy after the addition of the second one. Discontinuation of the therapy led to the disappearance of the side-effect. A tentative rechallenge with the same drug combination led to a second episode of acute severe encephalopathy.

**Conclusion:**

We speculate that this adverse reaction may be directly related to the effect of angiotensin II on the excretion of blood ammonia. Therefore, we suggest that patients with liver cirrhosis and portal hypertension are at risk of developing clinically relevant encephalopathy when angiotensin-converting enzyme inhibitor and angiotensin II receptor blocker combination therapy is administered, thus indicating the need for a careful clinical follow-up. In addition, the incidence of this serious side-effect should be rigorously evaluated in all patients with liver cirrhosis administered with this common treatment combination.

## Introduction

A combination therapy of angiotensin-converting enzyme inhibitors (ACEIs) and angiotensin II receptor blockers (ARBs) has been used to control proteinuria following initial demonstration of its efficacy [1]. However, recent concerns about the safety of this therapy have emerged, prompting several authors to urge for caution in its use [2]. In this case report, we describe the occurrence of a serious and unexpected adverse drug reaction after administration of the ACEI and ARB combination therapy to a patient with nephrotic syndrome and liver cirrhosis with severe portal hypertension. We suggest that the described adverse reaction is most likely related to the renal effects of the combination therapy and this should be taken into account in high-risk patients presenting with selected co-morbidities.

## Case presentation

A 40-year-old Caucasian man with liver cirrhosis and nephrotic syndrome, presented to our Liver Unit in December 2007. His liver disease had been diagnosed when he was 14 years old. Viral and autoimmune etiologies as well as inborn errors of metabolism were then excluded. After the occurrence of an episode of variceal bleeding at the age of 28, a successful prophylaxis of rebleeding with propranolol was started. From histological examinations, he had been diagnosed with hepatoportal sclerosis at age 30 and membranous glomerulonephritis at age 33 requiring the administration of furosemide (125 mg/day).

In January 2008, he was admitted as an inpatient to our Unit for a full evaluation for potential liver transplantation. He was asymptomatic and a physical examination revealed a slight hepatomegaly and splenomegaly, without asterixis, jaundice or ascites. An ultrasonography of our patient demonstrated evidence of portal hypertension, including an enlarged portal vein diameter and the presence of collateral circles. His proteinuria was 3.7 g/24 hours, despite the administration of losartan 50 mg/day prescribed six weeks previously, with normal creatinine values. Thus, ramipril 2.5 mg/day was added.

About 12 hours after the first dose of ramipril, our patient became unconscious. His Glasgow Coma Scale (GCS) was 6 (O1, V1, M4), blood pressure (BP) 130/80 and heart rate (HR) 60 bpm. No substantial changes from baseline were observed in biochemistry and blood gas analysis (Table 1); while his toxicological screening was negative. A cerebral computed tomography (CT) scan revealed no signs of compression or bleeding, while an electroencephalogram (EEG) showed overall slow brain activity compatible with toxic or metabolic alterations. His oral therapy was withdrawn and treatment with lactulose enema and intravenous hydration with branched-chain amino-acids was started, leading to recovery within 30-36 hours. After return to full consciousness, angiotensin block was re-introduced and after 48 hours his encephalopathy symptoms relapsed (GCS = 6). His CT scan was again negative and his EEG was similar to the previous one; while his blood ammonia concentration was dramatically elevated to 990 μg/dL. Our patient was admitted to the intensive care unit (ICU), where the episode was successfully treated.

**Table 1:** Blood exams and gas analysis upon admission and during the two episodes of encephalopathy

	Presentation	After 1st adverse event	After 2nd adverse event	Normal range
AST (U/L)	112	115	137	5-41
ALT (U/L)	71	81	84	7-41
Bilirubin (mg/dL)	1.7	2.8	2.7	<1
Creatinine (mg/dL)	0.8	0.9	0.8	0.7-1.3
Urea (mg/dL)	46	55	50	10-50
Albumin (g/dL)	1.5	1.6	1.6	3.6-4.8
Haemoglobin (g/dL)	9.4	11	10	13-17
WBC (10^3^/μL)	3.9	4.9	5.0	4.0-10.0
Platelets (10^3^/μL)	61	73	78	150.0-450.0
CRP (mg/L)	15	11	12	<5
PT (INR)	1.2	1.2	1.2	0.8-1.2
Na^+ ^(mEq/L)	139	139	140	135-145
K^+ ^(mEq/L)	4.7	5.0	4.2	3.6-5.2
pH	7.43	7.43	7.50	7.35-7.45
HCO_3 _(mmol/L)	23.6	22.1	24.6	21-23

AST: aspartate aminotransferase; ALT: alanine aminotransferase; WBC: white blood cell; PT: prothrombin time; INR: international normalized ratio; Na^+^: sodium, K^+^: potassium, HCO_3_: bicarbonate.

Angiotensin block was re-introduced for a third time with an addition of maximal lactulose therapy (oral and by enema) and oral rifaximin. By then, our patient was awake and conscious, although imperceptive and detached from his environment. After four days, ACEIs and ARBs were withdrawn, leading to a complete neurological recovery. He was discharged and remained asymptomatic with no further episodes of hepatic encephalopathy with low (normal) ammonia levels at repeated checks for 10 weeks.

## Discussion and conclusion

Targeting the renin-angiotensin system for BP control was introduced in the 1980s by the approval of the two main classes of drugs: ACEIs which are agents that hinder the conversion of angiotensin I (ATI) to the vasoactive angiotensin II (ATII), and ARBs which inhibit the ATI receptor involved in vasoconstriction, aldosterone secretion and sodium reabsorption. Both agents demonstrated similar efficacy profiles, leading to recent debates over the selection of initial antihypertensive medications [3].

Several clinical trials on humans were designed to investigate the effects of these agents, alone or in combination, in the control of hypertension [4,5], heart failure [6,7] and proteinuric kidney disease [2]. Most results confirmed that the ACEI and ARB combination therapy induced a slight improvement in hypertension control [4] and a definite reduction of proteinuria if concomitant renal damage was present [8,9]. However, increased incidences of hypotensive episodes [6,7], moderate to severe hyperkalemia [7] and adverse renal outcomes [2], the latter primarily reported in patients without proteinuria; have led to a reconsideration of the balance between the risks (increases of serum creatinine) and benefits (reductions of proteinuria) of the ACEI and ARB combination therapy. Renal side-effects, such as hyperkalemia and excessive reduction of the glomerular filtration rate, as well as potentially worse complications, such as acute renal failure, have to be further and more systematically evaluated. Thus, caution is advisable in the administration of this combination therapy until results from several ongoing trials with specific renal endpoints (Design of combination angiotensin receptor blocker and angiotensin-converting enzyme inhibitor for treatment of diabetic nephropathy (VA NEPHRON-D), Approaches to testing new treatments in autosomal dominant polycystic kidney disease: insights from the CRISP and HALT-PKD studies, and Protocol of the Long-term Impact of RAS Inhibition on Cardiorenal Outcomes (LIRICO) randomized trial)are available.

To the best of our knowledge, this is the first description of a serious, life-threatening adverse effect of the ACEI and ARB combination therapy, possibly connected to liver dysfunction in a patient with portal hypertension. We infer that the unfavorable reaction could be directly related to the effect of ATII on the excretion of blood ammonia, although the contribution of co-morbidity and multiple drug therapy cannot be ruled out.

**Figure 1:**
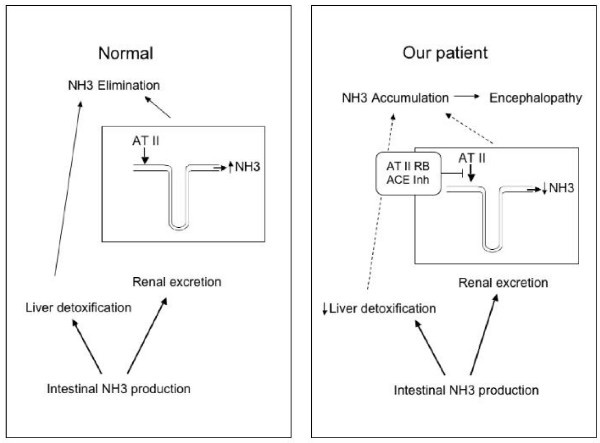
**In healthy controls the excretion of ammonia is mediated by two mechanisms: liver detoxification and renal excretion. Renal excretion is modulated by angiotensin II (ATII) at the level of the proximal tubule**. In our patient the hepatic mechanism is impaired, due to the liver cirrhosis, making the renal route essential for elimination of ammonia. Suppression of ATII activity through a combination of angiotensin-converting enzyme inhibitors and angiotensin II receptor blockers prevented adequate renal excretion, leading to an abrupt rise in serum ammonia concentration and the described neurological complications.

As previously described in animal models [10], ATII is essential to the control of ammonia production and excretion by the proximal tubule. Although the effects on serum ammonia levels of a pharmacological block by the renin-angiotensin system in humans are still undefined, our case suggests an overall reduction of renal excretion through the kidneys. In turn, this could have accounted for the abrupt rise in ammonia levels detected in a patient with increased susceptibility due to the concomitant liver cirrhosis and severe portal hypertension (Figure 1). We suggest a careful clinical follow-up and, possibly, monitoring of blood ammonia concentrations when ACEI and ARB combination therapy is administered to patients with liver cirrhosis and portal hypertension. The incidence of this serious side-effect associated with a common treatment should be rigorously evaluated in such high-risk patients.

## Consent

Written informed consent was obtained from the patient for publication of this case report and any accompanying images. A copy of the written consent is available for review by the Editor-in-Chief of this journal.

## Competing interests

The authors declare that they have no competing interests.

## Authors' contributions

SOP and AC were responsible for patient care and wrote the paper, MG suggested the pathogenetic mechanism and reviewed the paper, EV was responsible for patient care, MP edited and reviewed the paper and MZ reviewed the paper. All authors read and approved the final manuscript.
